# Fatty liver index is a predictor of incident diabetes in patients with prediabetes: The PREDAPS study

**DOI:** 10.1371/journal.pone.0198327

**Published:** 2018-06-01

**Authors:** Josep Franch-Nadal, Llorenç Caballeria, Manel Mata-Cases, Didac Mauricio, Carolina Giraldez-García, José Mancera, Albert Goday, Xavier Mundet-Tudurí, Enrique Regidor, for the PREDAPS Study Group

**Affiliations:** 1 redGDPS Foundation, Madrid, Spain; 2 Unitat de Suport a la Recerca Barcelona Ciutat, Institut Universitari d’Investigació en Atenció Primària Jordi Gol (IDIAP Jordi Gol), Barcelona, Spain; 3 Diabetes and Associated Metabolic Diseases Networking Biomedical Research Centre (CIBERDEM), Madrid, Spain; 4 Department of Medicine, University of Barcelona, Barcelona, Spain; 5 Liver and Digestive Diseases Networking Biomedical Research Centre (CIBEREHD), Madrid, Spain; 6 Unitat de Suport a la Recerca Barcelonès Nord, Institut Universitari d’Investigació en Atenció Primària Jordi Gol (IDIAP Jordi Gol), Barcelona, Spain; 7 Department of Endocrinology and Nutrition, Health Sciences Research Institute & University Hospital Germans Trias i Pujol, Badalona, Spain; 8 Preventive Medicine Service, University Hospital Infanta Elena, Madrid, Spain; 9 Preventive Medicine, Public Health and History of Science Department, Complutense University of Madrid, Madrid, Spain; 10 Health Center Ciudad Jardín, Málaga, Spain; 11 Endocrinology Service, Hospital del Mar, Barcelona, Spain; 12 Autonomous University of Barcelona, Bellaterra, Spain; 13 Epidemiology and Public Health Networking Biomedical Research Centre (CIBERESP), Madrid, Spain; 14 Health Research Institute, Hospital Clínico San Carlos (IdISSC), Madrid, Spain; The University of Hong Kong, HONG KONG

## Abstract

**Objectives:**

We evaluated the ability of the Fatty Liver Index (FLI), a surrogate marker of hepatic steatosis, to predict the development of type 2 diabetes (T2D) at 3 years follow-up in a Spanish cohort with prediabetes from a prospective observational study in primary care (PREDAPS).

**Methods:**

FLI was calculated at baseline for 1,142 adult subjects with prediabetes attending primary care centers, and classified into three categories: FLI <30 (no steatosis), FLI 30–60 (intermediate) and FLI ≥60 (hepatic steatosis). We estimated the incidence rate of T2D in each FLI category at 3 years of follow-up. The association between FLI and incident T2D was calculated using Cox regression models adjusted for age, sex, educational level, family history of diabetes, lifestyles, hypertension, lipid profile and transaminases.

**Results:**

The proportion of subjects with prediabetes and hepatic steatosis (FLI ≥60) at baseline was 55.7%. The incidence rate of T2D at 3 years follow-up was 1.3, 2.9 and 6.0 per 100 person-years for FLI<30, FLI 30->60 and FLI ≥60, respectively. The most significant variables increasing the risk of developing T2D were metabolic syndrome (hazard ratio [HR] = 3.02; 95% confidence interval [CI] = 2.14–4.26) and FLI ≥60 (HR = 4.52; 95%CI = 2.10–9.72). Moreover, FLI ≥60 was independently associated with T2D incidence: the HR was 4.97 (95% CI: 2.28–10.80) in the base regression model adjusted by sex, age and educational level, and 3.21 (95%CI: 1.45–7.09) in the fully adjusted model.

**Conclusions:**

FLI may be considered an easy and valuable early indicator of high risk of incident T2D in patients with prediabetes attended in primary care, which could allow the adoption of effective measures needed to prevent and reduce the progression of the disease.

## Introduction

Non-alcoholic fatty liver disease (NAFLD) is characterized by free fatty acid and triglyceride infiltration of hepatocytes that is not the result of significant alcohol intake or secondary to established liver diseases. It is an entity that encompasses a wide spectrum of lesions ranging from simple steatosis to steatohepatitis, and can progress to liver cirrhosis or even hepatocellular carcinoma [1, 2]. The estimated overall worldwide prevalence of NAFLD in the general adult population is about 25% [3], and increases substantially up to 40%-70% in subjects with established type 2 diabetes (T2D) [4]. In Spain, the prevalence of NAFLD has been estimated to be 26% among the adult population [5]. In addition, several epidemiological studies have consistently shown that NAFLD is an independent risk factor for incident T2D [6], metabolic syndrome [7], and cardiovascular disease (CVD) [8]. Given the progressive nature of the disease and the risk of serious consequences, health care providers are strongly advised to screen for NAFLD in all patients with T2D and to be more proactive in their management [9].

Liver biopsy is the gold standard method to diagnose NAFLD, and as it is quantitative, is the only one way to distinguish between simple steatosis and steatohepatitis. However, it has important limitations: it is an invasive procedure that carries an inherent risk to the patient, it is costly, and it is subject to high interobserver and sampling variability [10]. For these reasons, non-invasive methods, including imaging techniques or serum markers, are increasingly being used [11]. Abdominal ultrasound imaging is the method of choice because it is widely available and inexpensive, but in spite of being sensitive to detect fat accumulation when more than 33% of hepatocytes are steatotic, it does not provide information on the degree of fibrosis [11]. Furthermore, single serological markers are suboptimal for diagnosing NAFLD. Therefore, different models that combine multiple clinical and biochemical parameters have been proposed: these indices are easily obtained in routine clinical practice, are readily applicable, and some have shown good diagnostic accuracy for detecting steatosis [11, 12]. The Fatty Liver Index (FLI) is a simple algorithm that combines measures of triglycerides, gamma glutamyltranspeptidase (GGT), waist circumference and body mass index (BMI). FLI has an excellent discriminative ability to predict ultrasonographic fatty liver disease [13], and has been validated in both Asian and Western populations [14, 15]. FLI has been reported to correlate with insulin resistance, risk of coronary heart disease, risk of metabolic syndrome, early atherosclerosis, and rates of non-hepatic-related morbidity and mortality in nondiabetic subjects [16–19]. Finally, FLI also predicts incident hypertension in normotensive individuals [20], and further metabolic deterioration in women with previous gestational diabetes [21].

Two meta-analyses have reported that NAFLD diagnosed by either altered serum liver enzymes, radiological, or histological evidence increases the risk of developing T2D [7, 22], and FLI-diagnosed NAFLD has also been found to be a good predictor of incident T2D [23–26]. In addition, different studies have reported that ultrasonography-diagnosed NAFLD predicts the occurrence of prediabetes [27–31]. However, the risk of progression to T2D in subjects with prediabetes has not been studied at large, and all available studies so far have been conducted in Asian populations: two of them used ultrasonography to diagnose NAFLD [28, 32], and only one used FLI [33]. The possibility of using FLI in subjects with prediabetes is of great interest because, based on epidemiological and clinical evidence, people at increased risk of developing T2D should be the target of primary prevention efforts [34]. The objective of the present study was to determine the FLI's ability to predict the development of T2D in a primary care European population with prediabetes.

## Materials and methods

### Study design and data source

This was a follow-up study of a cohort of 1,184 subjects with prediabetes and another cohort of 838 subjects without alterations in glucose metabolism participating in the PREDAPS (Primary Health Care on the Evolution of Patients with Prediabetes) Study [35]. Briefly, the PREDAPS is a prospective observational study that collected baseline data from patients aged 30–74 years attending primary care centers in Spain throughout 2012 that are currently under follow-up to 5 years. Patients were excluded if they had any of the following conditions: diabetes, terminal disease, pregnancy, major surgery or hospital admission in the preceding 3 months, or hematological diseases that could interfere with the HbA1c value.

Prediabetes was defined using the American Association of Diabetes criteria for prediabetes [36], namely fasting plasma glucose (FPG) levels between 100 and 125 mg/dl and/or HbA1c between 5.7% and 6.4% (39 and 46 mmol/mol) in the prior 6 months. Recruited subjects were subsequently monitored annually to determine the incidence of new cases of T2D, defined as basal plasma glucose level ≥126 mg/dl on two occasions, or HbA1c ≥6.5% (≥48 mmol/mol) on two occasions, or both at the same time [36].

The present study analyzed the relationship between FLI and various clinical and sociodemographic characteristics measured at baseline and the onset of T2D up to the third year of follow-up among the cohort of subjects with prediabetes and available information (N = 1,142).

### Measures

Detailed information on the methodology of the study has been described elsewhere [35]. Clinical and demographic variables at baseline for this substudy included: age; sex; educational level; family history of diabetes; smoking status (classified as active smokers, ex-smokers, and never smokers), alcohol consumption (considering low-risk an intake ≤20 g/day in women and ≤40 g/day in men), physical activity (frequency and minutes per routine in the prior 2 weeks), sedentary lifestyle, defined as not following the World Health Organization recommendations on physical activity (i.e., at least 150 minutes a week of moderate aerobic activity, or 75 minutes a week of vigorous aerobic, or an equivalent combination of moderate and vigorous activities); and diet, based on the data collected on the frequency of consumption of fruits and vegetables, where subjects were classified into those who consumed those foods daily, and those who consumed them less frequently. Likewise, subjects were asked what they usually had for breakfast, and were divided into those who did not have breakfast or had an incomplete meal (only coffee, milk, chocolate, cocoa or yogurt), and those who had a proper breakfast.

During physical examination, height, weight, waist circumference and blood pressure were measured. Patients were classified with general obesity if their BMI was ≥30 kg/m^2^ and with abdominal obesity if waist circumference was ≥102 cm in men and ≥88 cm in women. Hypertension was defined as systolic blood pressure (SBP) ≥140 mmHg, diastolic blood pressure (DBP) ≥90 mmHg, currently taking antihypertensive drugs or personal history of high blood pressure.

The following 12-hour fasting plasma determinations were measured: FPG, HbA1c, lipid profile (total cholesterol [TC], high-density lipoprotein [HDL], low-density lipoprotein [LDL] and triglycerides [TG]), GGT and transaminases (aspartate transaminase [AST] and alanine transaminase [ALT]). Hypercholesterolemia was defined as total serum cholesterol ≥250 mg/dL, low HDL as <40 mg/dL in men and <50 mg/dL in women, high LDL ≥100 mg/dL, and hypertriglyceridemia as serum TG ≥150 mg/dL. Liver enzymes were considered elevated when GGT ≥40 U/L, or either AST or ALT ≥35 U/L. Subjects were considered to have metabolic syndrome when they met three or more of the following criteria: waist circumference >102 cm in men or >88 cm in women; TG ≥150 mg/dl; blood pressure ≥130/85 mmHg; HDL <40 mg/dl in men or <50 mg/dl in women; and FPG between 110 and 126 mg/dL [37].

The FLI as an indicator of hepatic steatosis was calculated based on the measures of TG, GGT, BMI and waist circumference, using the formula described in the literature [13]. Moreover, and as it has been established, FLI values (ranging from 0 to 100) were classified into three categories: <30, 30–59, and ≥60 [13]. FLI values of <30 and ≥60 would rule out and confirm the presence of hepatic steatosis, respectively.

The study was approved by the Ethics Committee of Institut Hospital del Mar. All patient records and information was anonymized and de-identified prior to analysis.

### Statistical analysis

We estimated the percentage and distribution of qualitative characteristics, and the mean of the quantitative characteristics for the three categories of FLI. Statistical significance was evaluated in the first case using the Chi-square test of heterogeneity, and in the second case with the analysis of variance (ANOVA) to test for differences between means. We calculated the incidence rate of diabetes per 100 person-years in each FLI category and then evaluated the association of the different baseline patient characteristics with the incidence of T2D. The measure of association was the hazard ratio (HR) and 95% confidence interval (CI) calculated by Cox regression models. We also estimated the degree to which the characteristics analyzed explained the association between FLI and the incidence of T2D. We first estimated a base model adjusted for age, sex and educational level, and then added the different characteristics to the baseline model, namely family history of diabetes, lifestyles, hypertension, lipid profile and transaminases. We did not include the variables involved in calculating the FLI (TG, GGT, BMI, and waist circumference). Patients who died (1.4%) and those who could not be followed up for 3 years (18.0%) were censored in the analysis, therefore they only contributed to the risk up to the date of death or loss to follow-up. We also conducted a sensitivity analysis excluding subjects with high-risk alcohol consumption. Finally, the extent to which FLI may be a better predictor of diabetes than insulin resistance is of interest. In the present study, fasting plasma insulin was not measured, although we estimated the predictive capacity of several surrogate markers that have shown high sensitivity and specificity for recognizing insulin resistance [38–45]. In particular, we analyzed the triglyceride glucose (TyG) index and various lipid ratios, namely the TG/HDL ratio, the total TC/HDL ratio, and the LDL/HDL ratio. Based on the value of each of these surrogate markers, subjects were stratified into tertiles. Statistical significance was established at a p-value <0.05. All analyses were conducted using the SPSS statistical package (version 20, SPSS, Chicago, Illinois, USA).

## Results

**Table 1** shows the sociodemographic and clinical characteristics collected at baseline according to FLI category. The proportion of subjects with prediabetes and hepatic steatosis (FLI ≥60), intermediate FLI (30–59), and no steatosis (FLI <30) was 55.7%, 27.9% and 16.4%, respectively. Subjects with FLI ≥60 were more frequently men, active smokers and high-risk drinkers, less frequently consumed fruits and vegetables daily, and exercised less regularly. Moreover, the frequency of metabolic syndrome was significantly higher in subjects with FLI ≥60 than in the other groups (56.3% vs. 8.6% in FLI < 30 and 21% in FLI 30–59; p<0.001), they also had higher rates of unfavorable body composition (higher BMI and waist circumference; p<0.001), adverse lipid profile (higher levels of TG and lower values of HDL cholesterol; p<0.001), and also higher blood pressure than the other FLI groups (p<0.001). Finally, subjects with FLI ≥60 also had higher glycemic levels (FPG and HbA1c; p<0.001 and p = 0.003, respectively), and their liver enzymes were more elevated than in the other groups (ALT, AST, and GGT; p<0.001).

**Table 1:** Sociodemographic, lifestyle, and clinical characteristics of subjects with prediabetes at baseline, according to baseline FLI.

	Fatty Liver Index			
	<30	30–59	≥60	P-value
**N (%)**	187 (16.4)	319 (27.9)	636 (55.7)	
**Gender, %**				
Men	19.8	51.1	58.8	<0.001
Women	80.2	48.9	41.2	
**Age, years, %**				
30–49	16.0	12.2	17.1	0.020
50–64	57.8	48.9	48.0	
65–74	26.2	38.9	34.9	
**Educational level, %**				
Less than secondary	58.8	67.4	64.0	0.15
Secondary or higher	41.2	32.6	36.0	
**Family history of diabetes, %**	44.9	48.6	46.5	0.71
**Smoking status, %**				
Active smoker	15.0	12.5	19.3	<0.001
Ex-smoker	28.3	37.9	42.0	
Never smoked	56.7	49.5	38.7	
**Alcohol consumption, %**				
None	42.7	35.1	30.2	0.003
Low risk	48.1	55.2	54.3	
High risk or harmful	9.2	9.7	15.6	
**Alcohol consumption (g/day), mean (SD)**	9.0 (24.3)	13.1 (34)	20.4 (50.7)	0.002
**Diet, %**				
Daily consumption of fruits	84.5	78.1	75.8	0.04
Daily consumption of vegetables	65.8	58.0	53.9	0.015
Complete breakfast	89.8	87.1	85.2	0.25
**Regular physical activity, %**	58.3	61.4	51.1	0.007
**Metabolic syndrome, %**	8.6	21.0	56.3	<0.001
**BMI (kg/m**^**2**^**), mean (SD)**	24.5 (2.4)	27.5 (2.3)	32.6 (4.2)	<0.001
**Waist circumference (cm), mean (SD)**	84.6 (7.3)	94.8 (6.4)	107.3 (9.6)	<0.001
**FPG (mg/dL), mean (SD)**	101.5 (11.7)	104.7 (10.7)	106.8 (9.9)	<0.001
**HbA1c (%), mean (SD)**	5.8 (0.3)	5.8 (0.3)	5.9 (0,3)	0.003
**SBP (mmHg), mean (SD)**	127.9 (14.8)	132.7 (14.7)	137.7 (16.3)	<0.001
**DBP (mmHg), mean (SD)**	77.0 (8.6)	79.7 (8.6)	82.9 (9.4)	<0.001
**AST (U/L), mean (SD)**	20.5 (7.4)	22.8 (9.3)	25.3 (11.3)	<0.001
**ALT (U/L), mean (SD)**	20.4 (9.6)	24.4 (11.3)	30.8 (18.0)	<0.001
**GGT (U/L), mean (SD)**	18.5 (8.6)	26.5 (14.8)	44.7 (41.3)	<0.001
**Total cholesterol (mg/dL), mean (SD)**	212.9 (35.6)	209.3 (39.7)	208.8 (37.6)	0.41
**HDL cholesterol (mg/dL), mean (SD)**	63.2 (16.1)	56.2 (13.2)	50.6 (13.0)	<0.001
**LDL cholesterol (mg/dL), mean (SD)**	132.8 (32.9)	129.7 (35.5)	126.9 (34.1)	0.10
**Triglycerides (mg/dL), mean (SD)**	85.2 (30.7)	117 (45.9)	156.4 (83.1)	<0.001

ALT, alanine transaminase; AST, aspartate transaminase; BMI, body mass index; DBP, diastolic blood pressure; FPG, fasting plasma glucose; GGT, gamma glutamyltranspeptidase; HDL, high density lipoprotein; LDL, low density lipoprotein; SBP, systolic blood pressure; SD, standard deviation

### Incidence of diabetes over 3 years and associated risk factors

Subjects were followed during an average of 2.83 years (SD = 0.87), and 107 out of the 1,142 initially assessed (9.4%) developed T2D at the end of follow-up. By FLI category, the proportion of participants who developed T2D was the highest among those with FLI ≥60 (n = 107; 16.8%), followed by those with FLI 30–59 (n = 27; 8.5%) and those with FLI <30 (n = 7; 3.7%). This corresponded to a T2D incidence rate of 1.3, 2.9 and 6.0 per 100 person-years in the categories FLI<30, FLI 30–59 and FLI ≥60, respectively.

Among all sociodemographic and lifestyle variables, only family history of T2D was found as a significant risk factor for incident diabetes, while daily consumption of fruit was the only significant protective factor (**Table 2**). Regarding clinical and biochemical parameters, the most significant variables increasing the risk of developing T2D were metabolic syndrome (HR = 3.02; 95%CI = 2.14–4.26) and FLI ≥60 (HR = 4.52; 95%CI = 2.10–9.72). Other individual risk factors moderately increasing the likelihood of progression were general obesity based on BMI, abdominal obesity based on waist circumference, presence of hypertension, high triglycerides, and elevated liver enzymes. Conversely, high levels of HDL cholesterol were protective against incident T2D.

**Table 2:** Bivariate analysis of baseline characteristics and incidence of diabetes in patients with prediabetes at 3 years follow-up.

Variable	HR (95%) CI)
**Gender**	
Men	1.00
Women	1.01 (0.73–1.41)
**Age**	
30–49 years	1,00
50–64 years	0.93 (0.58–1.47)
65–74 years	0.94 (0.58–1.54)
**Educational level**	
Less than secondary	1.00
Secondary or higher	0.88 (0.62–1.25)
**Family history of diabetes**	
No	1.00
Yes	1.58 (1.13–2.21)
**Tobacco consumption**	
Smoker	1.00
Ex-smoker	1.49 (0.89–2.48)
Never smoker	1.16 (0.69–1.95)
**Alcohol consumption**	
None	1.00
Low risk	0.74 (0.52–1.07)
High risk or harmful	0.85 (0.51–1.43)
**Daily consumption of fruits**	
No	1.00
Yes	0.57 (0.40–0.81)
**Daily consumption of vegetables**	
No	1.00
Yes	1.04 (0.75–1.46)
**Complete breakfast**	
No	1.00
Yes	0.71 (0.46–1.09)
**Regular physical activity**	
No	1.00
Yes	0.74 (0.53–1.03)
**BMI (kg/m**^**2**^**)**	
<30	1.00
≥30	1.80 (1.29–2.51)
**Abdominal circumference (cm)**	
<88 cm (women)/ <102 cm (men)	1.00
≥88 cm (women)/ ≥102 cm (men)	2.21 (1.44–3.38)
**Blood pressure (mmHg)**	
<140/90	1.00
≥140/90	1.58 (1.13–2.20)
**Liver enzymes (U/L)**	
AST ≤35	1.00
AST >35	2.18 (1.39–3.41)
ALT ≤35	1.00
ALT >35	1.93 (1.34–2.76)
GGT ≤40	1.00
GGT >40	1.66 (1.18–2.35)
**Lipid profile (mg/dL)**	
Total cholesterol <250	1.00
Total cholesterol ≥250	0.85 (0.51–1.42)
HDL cholesterol <40 (men)/ <50 (women)	1.00
HDL cholesterol ≥40 (men)/ ≥50 (women)	0.58 (0.41–0.82)
LDL cholesterol <100	1.00
LDL cholesterol ≥100	0.87 (0.58–1.30)
Triglycerides <150	1.00
Triglycerides ≥150	1.63 (1.16–2.30)
**Metabolic syndrome**	
No	1.00
Yes	3.02 (2.14–4.26)
**Fatty liver index**	
<30	1.00
30–60	2.22 (0.97–5.11)
≥60	4.52 (2.10–9.72)

ALT, alanine transaminase; AST, aspartate transaminase; BMI, body mass index; CI, confidence interval; DBP, diastolic blood pressure; FPG, fasting plasma glucose; GGT, gamma glutamyltranspeptidase; HDL, high density lipoprotein; HR, hazard ratio; LDL, low density lipoprotein; SBP, systolic blood pressure

### FLI for the prediction of incident T2D in prediabetes

Multivariate models for the development of T2D at 3 years of follow-up in patients with prediabetes (**Table 3**) showed that the presence of FLI ≥60 was in all cases a significant independent risk factor. In the base model, adjusted for age, sex and educational level, the HR in the category FLI ≥60 with respect to FLI <30 was 4.97 (95%CI = 2.28–10.80). When the baseline model was adjusted by five other characteristics separately, the HR decreased slightly (HRs between 4.13 and 4.82). Adjustment for all the variables combined decreased the magnitude of the HR to 3.21 (95%CI = 1.45–7.09). In the sensitivity analysis restricted to subjects without high-risk alcohol consumption, the magnitude of the association increased: the HR for FLI ≥60 was 5.09 (95%CI = 2.20–11.76) in the base model, and 3.54 (95%CI = 1.51–8.31) in the fully adjusted model.

**Table 3:** Hazard ratios and 95% confidence intervals of multivariate models for the risk of incident T2D in patients with prediabetes at 3 years of follow-up according to FLI categories.

	Fatty Liver Index		
	<30	30–59	≥60
Incident T2D; unadjusted	1.00	2.22 (0.97–5.11)	4.52 (2.10–9.72)
Base model: Incident T2D adjusted for age, sex and educational level	1.00	2.40 (1.03–5.55)	4.97 (2.28–10.80)
Base model adjusted for family history of T2D	1.00	2.31 (1.00–5.36)	4.82 (2.22–10.48)
Base model adjusted for lifestyle*	1.00	2.26 (0.97–5.24)	4.63 (2.12–10.10)
Base model adjusted for hypertension	1.00	2.30 (0.99–5.34)	4.59 (2.10–10.03)
Base model adjusted for lipids (total and HDL cholesterol)	1.00	2.35 (1.01–5.44)	4.58 (2.09–10.01)
Base model adjusted for transaminases (AST, ALT)	1.00	2.22 (0.96–5.14)	4.13 (1.88–9.04)
Base model adjusted for family history of T2D, lifestyle* hypertension, lipids and transaminases			
All	1.00	1.96 (0.85–4.54)	3.21 (1.45–7.09)
Men	1.00	1.53 (0.43–5.40)	1.70 (0.50–5.74)
Women	1.00	1.73 (0.53–5.59)	4.95 (1.73–14.29)

*Includes tobacco consumption, alcohol intake, consumption of fruits and vegetables, breakfast and physical activity

T2D, type 2 diabetes mellitus; ALT, alanine transaminase; AST, aspartate transaminase; HDL, high density lipoprotein

Finally, **Table 4** shows the findings on the models for the risk of development of T2D at 3 years of follow-up in patients with prediabetes according to tertiles of surrogate markers for insulin resistance. After adjusting for all variables, the magnitude of the hazard ratios for all markers was lower than that observed with FLI.

**Table 4:** Hazard ratios and 95% confidence intervals for the risk of incident T2D in patients with prediabetes at 3 years of follow-up according to tertiles of surrogate markers of insulin resistance.

Surrogate markers of insulin resistance	Bivariate analysis			Multivariate analysis^1^		
	HR	95% CI		HR	95% CI	
**TyG Index**						
1^st^ tertile	1.00			1.00		
2^nd^ tertile	1.24	0.71	2.16	1.00	0.56	1.78
3^rd^ tertile	2.44	1.47	4.05	1.87	1.09	3.21
**TG/HDL Ratio**						
1^st^ tertile	1.00			1.00		
2^nd^ tertile	1.35	0.83	2.17	1.10	0.66	1.82
3^rd^ tertile	1.95	1.25	3.04	1.59	0.94	2.68
**TC/HDL Ratio**						
1^st^ tertile	1.00			1.00		
2^nd^ tertile	1.40	0.89	2.20	1.26	0.78	2.02
3^rd^ tertile	1.73	1.13	2.66	1.40	0.82	2.37
**LDL/HDL Ratio**						
1^st^ tertile	1.00			1.00		
2^nd^ tertile	1.20	0.78	1.85	1.07	0.69	1.68
3^rd^ tertile	1.36	0.90	2.06	1.13	0.69	1.83

^1^Adjusted for age, sex and educational level, family history of T2D, tobacco consumption, alcohol intake, consumption of fruits and vegetables, breakfast and physical activity, hypertension, lipids and transaminases.

CI, confidence interval; HDL, high density lipoprotein; HR, hazard ratio; LDL, low density lipoprotein; TC, total cholesterol; triglyceride glucose index (TyG index)

## Discussion

The results of the present prospective study conducted in Spain in a cohort of patients with prediabetes (PREDAPS study) showed that FLI-diagnosed hepatic steatosis is associated with a risk of developing T2D at 3 years of follow-up. Moreover, this association was independent of possible confounding factors such as family history of diabetes, lifestyle, hypertension, lipid profile and level of transaminases. The only available study performed so far to assess the incidence of T2D in subjects with prediabetes and NAFLD diagnosed through FLI was performed in a Japanese population [33]. However, new T2D was diagnosed based on self-administered questionnaires, and it is not clear whether the results could be as well applied to Western or Caucasian populations [33]. In our study, the diagnosis of T2D was based on basal plasma glucose or HbA1c values, and it is the first study evaluating the ability of the FLI to predict diabetes in subjects with prediabetes according to ADA criteria [36].

In the PREDAPS study, the baseline prevalence of hepatic steatosis (i.e., FLI ≥60) was 56%, which is higher than the 19% reported in the Japanese study [33], closer to the approximately 22–40% reported by studies with ultrasonography-diagnosed NAFLD [27, 28, 30, 46–49], and lower than the 75% observed in overweight and obese subjects in a study using magnetic resonance (MR) [50].

In a previous study conducted in France in subjects from the general population, the authors found that FLI ≥70 was associated with the development of T2D [23]. Our study shows that an FLI ≥60 can be a good predictor of T2D in subjects with impaired glucose metabolism. An important observation in the present study was that patients with FLI ≥60 had the most altered metabolic profile, with a high prevalence of metabolic syndrome and high values of each of its constituent components. These results are in line with the study of Nishi et al. conducted in Japanese subjects with prediabetes [33] and also with those of a large population-based study conducted in Italy in patients with established T2D [51]. These findings are not surprising if we take into account that the degree of liver fat content correlates with all components of metabolic syndrome [52]. This correlation can be attributed to the fact that both NAFLD and T2D share a series of common physiopathological mechanisms, including alterations in glucose and lipid metabolism, insulin resistance, and environmental and genetic factors [51, 53].

Another interesting finding was that patients with FLI≥60 had significantly higher mean levels of transaminases than the lower FLI groups, although over half of them exhibited normal values. It has been previously reported that a substantial number of patients with NAFLD, even in those with a more advanced stage of the disease, have normal transaminases [54–56]. This is important, since clinicians often rely on abnormal transaminases to identify patients with NAFLD, but it has been shown that an increase, even when levels are within the normal range, is an independent predictor of incident T2D and metabolic syndrome [57–59].

Only two previous studies conducted in Asian populations have assessed the risk of progression to T2D from prediabetes using ultrasonography to diagnose NAFLD. In one prospective study conducted in Japan, prediabetes was found as the strongest predictor for the development of T2D at 10-years follow-up, with a 6.4 fold-risk compared with subjects with normal glucose [28]. In another study, conducted in Korea, the risk for incident T2D at 5 years follow-up was enhanced by 9-fold only in subjects with NAFLD and concomitant impaired fasting glucose, but no increased risk of T2D was observed among those with NAFLD and normal glucose [30]. In our study, 9.4% of patients with prediabetes progressed to T2D during the 3-years follow-up, and the risk of progression was 3.21 times higher for subjects with FLI ≥60 than in those with FLI <30 in the fully adjusted model. This is in agreement with the results obtained in the only available and comparable study using FLI to diagnose NAFLD [33]. In the Nishi et al. Japanese study [33], 11.5% of men and 6% of women with prediabetes developed T2D during 3 years follow-up, and they observed similar risk figures after adjusting for almost the same variables as in our case (odds ratio 2.68 in men and 10.35 in women), although in our study the magnitude of HR was lower (1.70 in men and 4.95 in women).

FLI could be of use in routine clinical practice as an additional screening tool to identify those with prediabetes at high risk of progression that would benefit from early interventions [9, 60]. For instance, weight loss via energy restriction or physical activity have been shown to reduce liver fat and improve hepatic glucose metabolism within weeks [61, 62], and the resolution of fatty liver to significantly reduce the risk of T2D development to a level similar to individuals without NAFLD [63, 64]. Moreover, diabetes is an independent factor of NAFLD progression and of the development of cirrhosis [65–67], and a recent study showed that lipid-lowering therapy with statins correlates with improvement in the FLI score [19], and another one that long-term pioglitazone treatment in patients with prediabetes or T2D led to marked histologic improvements in hepatic steatosis, inflammation, and ballooning without worsening fibrosis [68]. Finally, NAFLD is an independent risk factor for the development of micro and macrovascular complications in patients with diabetes [69, 70], and statins have also been shown to substantially reduce cardiovascular morbidity and mortality by >50% compared with high-risk patients with normal liver structure and function [71].

The present study has both strengths and limitations that should be acknowledged. Among its strengths is its prospective design, which included measurement of a large variety of possible confounding factors. However, given the complex bidirectionality between NAFLD, insulin resistance and hyperglycemia, it is challenging to distinguish whether NAFLD is a cause or a consequence of insulin resistance and T2D. With regard to limitations, it must be noted that the different parameters that make up the FLI are also risk factors for T2D, which could call into question whether NAFLD based on the FLI is an independent predictor for the presence of T2D.

Another limitation was that we diagnosed NAFLD with FLI instead of liver biopsy. On the one hand, FLI was found to be highly reliable in a European study when subsequently confirmed by abdominal ultrasound [15], although another population study, where the diagnosis was confirmed by magnetic resonance spectroscopy, found a more moderate diagnostic precision [12]. On the other hand, FLI does not give information on the severity of hepatic steatosis, and we could not assess whether the presence of more severe forms of the disease (i.e., fatty infiltration plus inflammation [NASH]) appears to afford a greater risk for incident T2D than simple steatosis, as previously described [72]. Moreover, patients with FLI ≥60 were more likely to report high-risk alcohol, and GGT, an analytical marker of alcohol consumption, was also higher than in the lower FLI groups. It is known that alcohol consumption, regardless of the amount, can cause hepatic steatosis. Thus, although we cannot rule out a possible classification bias, whereby fatty liver in some patients classified as having NAFLD was actually due to alcohol consumption, the results were unchanged when subjects with high-risk alcohol intake were excluded from the analysis. In addition, we relied on FPG and/or HbA1c to diagnose diabetes and prediabetes, and the lack of a 2-hour oral glucose tolerance test might have resulted in the inclusion of subjects with undiagnosed diabetes at baseline or underestimated the number of patients with incident T2D cases. Lastly, a potential weakness of the study is that we had no data on insulin resistance (i.e., HOMA-IR index or fasting insulin), which is strongly associated with NAFLD and may contribute to the development of T2D [32, 73]. However, we were instead able to calculate several surrogate markers of insulin resistance. The magnitude of HR that evaluated the relationship between these markers and development of T2D at 3 years of follow-up was lower than that the one obtained in subjects whose FLI was ≥60. On the other hand, although high values of FLI are associated with reduced insulin sensitivity [17, 26], fatty liver diagnosed as FLI ≥60 has been shown to be a predictor of incident diabetes independently of insulin resistance [24]. In addition, insulin resistance did not significantly contribute to reduce the risk when it was considered as a confounding factor [23, 26].

## Conclusions

FLI should be considered an easy and valuable tool to be used in primary care routine clinical practice for early identification of significant liver disease in subjects with prediabetes and to stratify the risk of developing T2D. This way, those at the greatest risk could be carefully monitored so that effective interventions to prevent and reduce progression of the disease could be adopted.
